# Advancements in gene therapies targeting mutant KRAS in cancers

**DOI:** 10.1007/s10555-025-10243-9

**Published:** 2025-01-17

**Authors:** Yuhang Wang, Thuy Anh Bui, Xinpu Yang, Gyorgy Hutvagner, Wei Deng

**Affiliations:** 1 School of Biomedical Engineering, University of Technology Sydney, Ultimo, NSW 2007 Australia; 2 Ingham Institute for Applied Medical Research, 1 Campbell St, Liverpool, NSW 2170 Australia; 3 School of Clinical Medicine, Faculty of Medicine, University of New South Wales, Kensington, NSW 2052 Australia; 4 Graduate School of Biomedical Engineering, University of New South Wales, Kensington, NSW 2052 Australia

**Keywords:** KRAS mutation; Cancer treatment, Gene therapy, Inhibitor; Lipid nanocarrier

## Abstract

Mutations in the KRAS gene are well-known tumourigenic drivers of colorectal, pancreatic and lung cancers. Mechanistically, these mutations promote uncontrolled cell proliferation and alter the tumour microenvironment during early carcinoma stages. Given their critical carcinogenic functions, significant progress has been made in developing KRAS inhibitors for cancer treatment. However, clinical applications of these KRAS inhibitor compounds are limited to specific cancer types which carry the relevant KRAS mutations. Additionally, clinical findings have shown that these compounds can induce moderate to serious side effects. Therefore, new approaches have emerged focusing on the development of universal therapeutics capable of targeting a wider range of KRAS mutations, minimising toxicity and enhancing the therapeutic efficacy. This review aims to examine these therapeutic strategies in the context of cancer treatment. It firstly provides an overview of fundamental KRAS biology within the cell signalling landscape and how KRAS mutations are associated with cancer pathogenesis. Subsequently, it introduces the development of current KRAS inhibitors which target certain KRAS mutants in different types of cancer. It then explores the potential of gene therapy approaches, including siRNA, miRNA and CRISPR methodologies. Furthermore, it discusses the use of lipid-based nanocarriers to deliver gene cargos for targeting KRAS gene mutants. Finally, it provides the insights into the future prospects for combatting KRAS mutation-associated cancers.

**Graphical Abstract:**

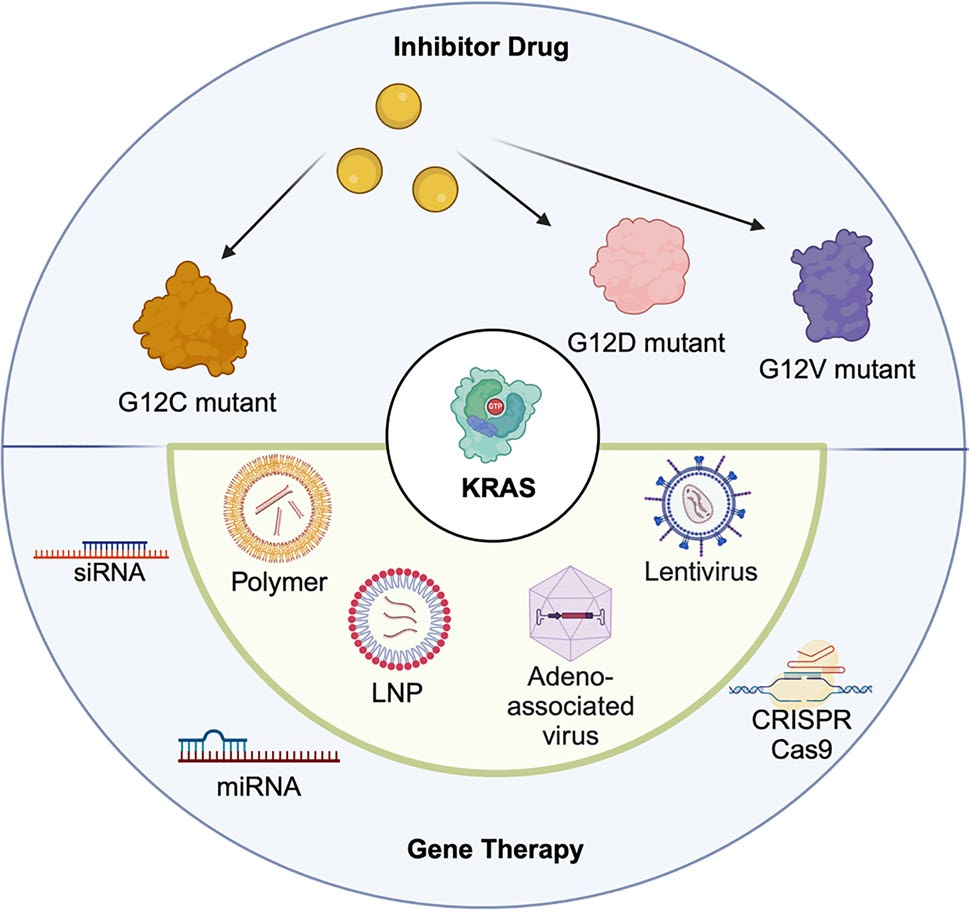

## Structure and function of KRAS

**Fig. 1:**
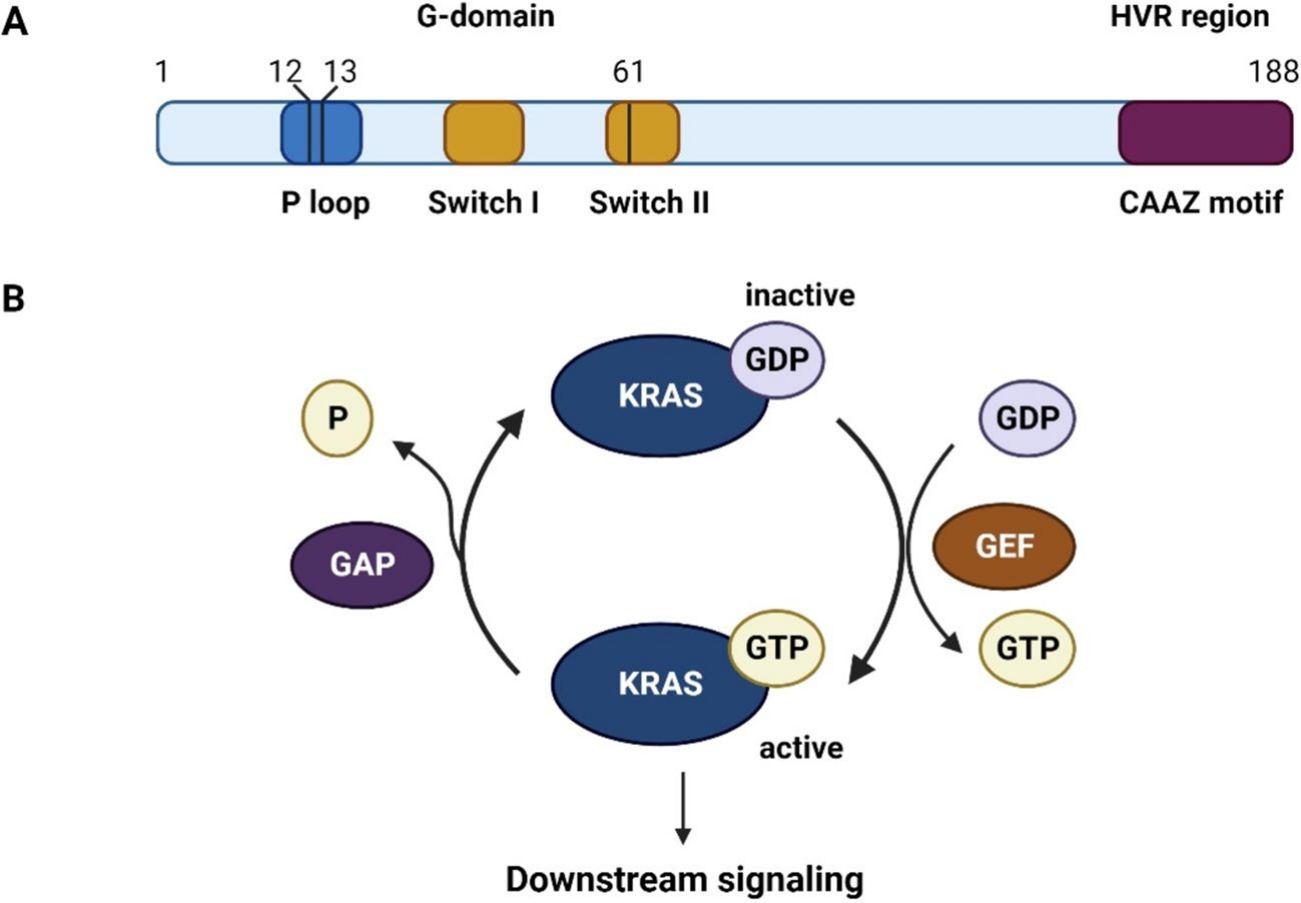
KRAS structure and function. **A** KRAS protein’s functional domains: the G domain housing essential signalling regions and the HVR domain necessary for membrane localization; **B** KRAS functions as a binary switch in signal transduction, transitioning between inactive GDP-bound and active GTP-bound states. Its activity is regulated by interactions with GAP and GEF

The KRAS gene, part of the rat sarcoma viral oncogene family, was identified in 1982, along with HRAS and NRAS [1, 2]. This gene is located on chromosome 12 and produce two protein variants, KRAS-4A and KRAS-4B with the latter predominates in cells [3]. At protein level, KRAS acts as a membrane-bound G protein which functions as a binary switch between its inactive GDP-bound and active GTP-bound states to modulate signal transduction from activated membrane receptors to downstream signalling pathways within the target cells [4, 5]. To perform this function, the KRAS polypeptide consists of six beta strands and five alpha helices with specific regions such as P-loop, Switch I and Switch II which regulate KRAS molecular activity and interactions with other cellular components (Fig. 1A) [5]. With its low intrinsic GTPase activity, KRAS relies on GTPase activating proteins (GAPs) like SOS and NF1 to catalyse hydrolysis (Fig. 1B) [6]. Mechanistically, under normal conditions, KRAS remains inactive, bound to GDP [7]. At the presence of growth factors, stimulated cells initiate the substitution of GDP by GTP, thus activating KRAS and initiating signalling cascades [8]. After that, GAPs promote KRAS inactivation by enabling GDP binding.

**Fig. 2:**
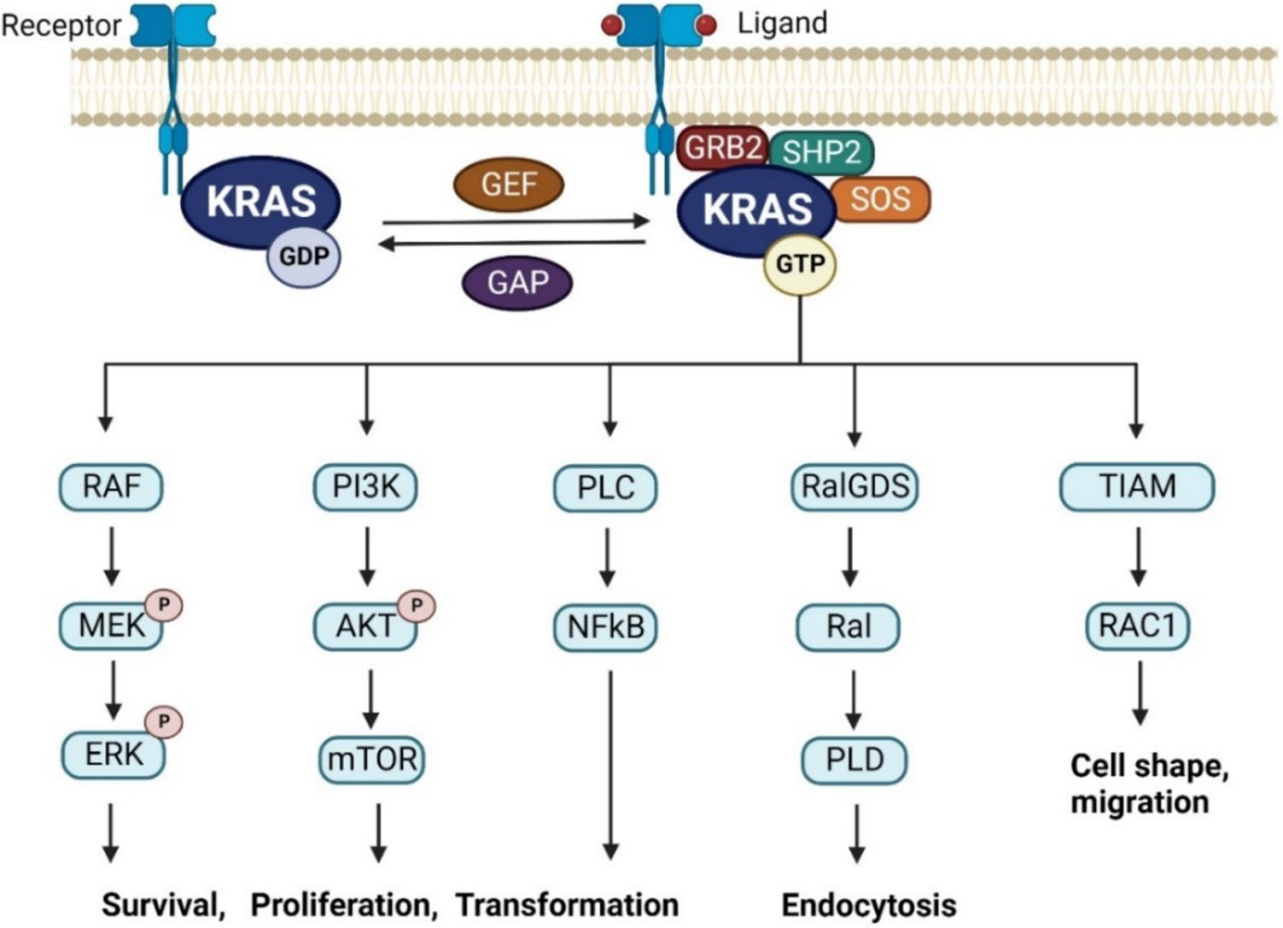
KRAS upstream and downstream signalling pathways. KRAS receives signals from various receptors, including receptor tyrosine kinases, leading to the activation of downstream effectors such as RAF, MEK and ERK in the MAPK pathway, as well as PI3K/AKT/mTOR pathway. KRAS mutations lock itself in sustained “active state”, which continuously sending stimulations to all of KRAS downstream signalling pathways, resulting in dysregulated cell proliferation, survival and differentiation, contributing to tumourigenesis, cancer progression and modifications of tumour microenvironment

KRAS activation requires key components such as GRB2-SOS1, RAS-GRF1 and Src homology phosphatase 2 (SHP2) (Fig. 2) [9]. The GRB2-SOS1 complex plays a crucial role, acting as an intermediary activated by growth factors like epidermal growth factor (EGF), platelet-derived growth factor (PDGF) and fibroblast growth factors (FGFs) [9]. Upon receptor tyrosine kinase activation, GRB2 binds to phosphorylated receptors and recruits SOS1, a guanine nucleotide exchange factor (GEF) [10]. This leads to the activation of KRAS by catalysing the exchange of GDP for GTP. RAS-GRF1, another GEF, operates in the brain and activates KRAS in mature neurons, with its efficacy enhanced by higher Ca2 + concentrations and phosphorylation by protein kinase A [10]. SHP2, a protein tyrosine phosphatase, plays a crucial role in KRAS activation by facilitating GRB2-SOS1 complex recruitment and dephosphorylating substrates to positively impact KRAS activation [11, 12]. This pathway also modulates negative regulators and other signalling molecules in the KRAS pathway [13, 14].

Once activated, KRAS can regulate a range of cellular responses via different downstream signalling pathways such as the RAF-MEK-ERK pathway, PI3K pathway and other pathways (Fig. 2). Firstly, the RAF-MEK-ERK pathway is activated when KRAS recruits RAF to the plasma membrane, then initiating a phosphorylation cascade that regulates cell proliferation, differentiation, migration and other vital activities [15, 16]. In the PI3K-AKT-mTOR pathway, KRAS activates PI3K, leading to AKT phosphorylation and subsequent modulation of cell proliferation, apoptosis and metabolism [17]. Other signalling pathways include RAL guanine nucleotide dissociation stimulator (RalGDS), influencing cellular processes through RAL proteins, TIAM1, RAC1-specific guanine nucleotide exchange factors, and the phosphatidylinositol signalling pathway by activating PLCε [18, 19]. These pathways also contribute to the control of cellular activities such as proliferation, differentiation and migration [20].

## KRAS mutations in cancer pathogenesis

As aforementioned, wild-type KRAS (WT KRAS) is crucial for cell signalling and homeostasis [21]. In the context of cancer, the WT KRAS gene also serves as a reference for normal cellular function and even acts as a tumour suppressor [22]. However, KRAS mutations, primarily single-base missense mutations occurring at codons 12, 13 or 61, have been found to drive tumourigenesis of pancreatic ductal adenocarcinoma (PDAC), colorectal cancer (CRC) and non-small-cell lung cancer (NSCLC) [23]. In PDAC, a positive association of KRAS mutations has been found with a mutation rate of 67.61%. Among these mutations, the most prevalent subtype is G12D, constituting 26.84% of the total mutations in this cancer. Similar KRAS mutation prevalence has been shown in CRC, with a total mutation rate of 35.77% and the dominant subtype G12D representing 9.87% of mutations [24]. The third highest incidence of KRAS mutations has been found in NSCLC, with a total mutation rate of 20.42%, and G12C mutation being the most frequent subtype, representing 8.38% of cases [25]. These mutations commonly result in the presence of a constitutive active KRAS mutant protein, thus leading to uncontrolled cell growth, tumour formation and resistance to specific cancer treatments [26]. Additionally, the simultaneous occurrence of KRAS mutations with other co-mutations can also influences both KRAS functionality and tumour progress [29]. For example, in NSCLC with KRAS mutations, STK11, KEAP1 and TP53 mutations have been detected where TP53 mutations account for around 39.4% of KRAS-mutation cancer cases.

Apart from driving tumourigenesis, mutant KRAS can also affect the tumour microenvironment (TME) by regulating cytokine release, recruitment of immune cells to the tumour sites for enhanced inflammatory responses as well as facilitating immune escape [27, 28]. This phenomenon has been observed in pancreas, colon and lung cancers harbouring KRAS mutations [2]. Mechanistically, the oncogenic KRAS variant activates inflammatory cytokines, chemokines and downstream signalling pathways to promote tumour development and invasiveness [29, 30]. For instance, KRAS overactivation in pancreatic cancer increases interleukin-6 (IL-6) secretion, which facilitates tumour development via the JAK1/STAT3 pathway and triggers reactive oxygen species generation and oxidative stress responses [31]. In lung cancer and PDAC, KRAS mutations can directly induce interleukin-8 production and secretion by tumour cells, thus triggering endothelial cell recruitment, tumour-associated inflammation and angiogenesis [32, 33]. Furthermore, the presence of KRAS/STK11 co-mutations or KRAS/P53 co-mutations has been linked to a TME abundant in CD8 + tumour-infiltrating lymphocytes and activated dendritic cells [34].

## Current inhibitor strategies targeting mutant KRAS

Given the crucial role of mutant KRAS in tumour initiation and progression, the development of therapeutics specifically targeting KRAS mutants hold great potential for tumour suppression. This section explores how KRAS inhibitors have been developed to target G12C and other mutant forms of KRAS in specific types of cancer.

### Clinical development of KRAS G12C inhibitors

Among the main KRAS mutation sites, G12C mutation is distinct from G12D and G12V in its ability to alternate interactions with downstream effectors, cycling between GDP-bound and GTP-bound states [35]. In the KRAS G12C mutant, the proximity of a novel cysteine residue adjacent to switch II facilitates the binding of potential inhibitors with cysteine solely through disulphide bonds, but only when KRAS is in the inactive GDP-bound state [35, 36]. This unique characteristic of KRAS G12C enables it to be targeted and stabilised in an inactive state by inhibitors interacting with cysteine residues, presenting a promising avenue for effective therapeutic interventions [37]. Originating from Shokat and colleagues’ work in 2013, the concept of using cysteine residues in the KRAS G12C mutant to create specific covalent inhibitors involved screening a compound library with protein mass spectrometry, specifically for KRAS G12C in its GDP state [36]. These G12C inhibitors can disrupt both switch regions of the mutant KRAS while sparing the WT KRAS, thus altering KRAS’s nucleotide preference to favour GDP, hindering its binding to Raf [35].

So far, several KRAS G12C inhibitors, including AMG510 (Sotorasib), MRTX849, LY3537982, GDC-6036 and D-1553, have gained approval for cancer treatment. AMG510, the first-in-class small molecule KRAS G12C inhibitor, irreversibly binds to Cys12, inducing an inactive status of this protein [38]. Phase I clinical study showed 32.2% objective response rate (ORR), 88.1% disease control rate (DCR), and 6.3 months median progression-free survival [39]. Phase II study confirmed efficacy with 37.1% ORR and 80.6% DCR [48]. In Phase III, AMG510 improved progression-free survival vs. docetaxel but did not significantly enhance overall survival [40]. In 2021, AMG510 became the first approved treatment for KRAS G12C-mutant non-small cell lung cancer (NSCLC) post-prior systemic therapy [40].

MRTX849 (Adagrasib) is another irreversible covalent KRAS G12C inhibitor [41]. Compared to AMG510, MRTX849 exhibits a 24-h half-life and wider tissue distribution. In the phase I/II KRYSTAL-1 study on 116 cancer patients carrying KRAS G12C mutations, 42.9% demonstrated confirmed objective responses, with a median duration of 8.5 months, progression-free survival of 6.5 months, and overall survival reaching 12.6 months [42]. Despite a high rate of treatment-related adverse events (97.4%, 44.8% grade 3 or higher, mainly gastrointestinal), manageable through dose adjustments, the discontinuation rate was 6.9% [41, 43, 44]. Additionally, MRTX849’s ability to penetrate blood–brain barrier and exhibit efficacy against brain metastases led to FDA accelerated approval for treating advanced KRASG12C-mutated NSCLC after first-line standard care, aligning with AMG510’s approval level [45].

LY3537982, another KRAS G12C inhibitor with a lower IC50 compared to both AMG510 and MRTX849, demonstrates potent anti-tumour efficacy, including complete regression in KRAS G12C tumours [46, 47]. In a Phase I clinical trial with doses ranging from 50 to 200 mg twice daily, LY3537982 showed a promising safety profile for KRAS G12C—related cancer [48]. Notably, LY3537982 was well-tolerated by patients who had previously shown intolerance to other KRAS G12C inhibitors [48]. The treatment-emergent adverse events (TEAEs) observed in over 10% of patients were mostly of grade 1 severity, including diarrhoea, constipation, fatigue, peripheral oedema and nausea [48]. Neutropenia was reported in one patient, and importantly, no treatment-related adverse events or deaths were recorded in this trial [48].

GDC-6036 (Divarasib), another covalent KRAS G12C inhibitor, is ongoing investigated in phase 1 clinical trial for various solid tumours as both monotherapy and in combination with other anti-cancer therapies [46]. In a phase I trial using GDC-6036 as single agent in solid tumours, it has been reported with a confirmed response rate of 53.4% in NSCLC patients, and a median progression-free survival of 13.1 months [49]. Among CRC patients, the confirmed response rate was 29.1%, and the median progression-free survival was 5.6 months [49]. Treatment-related adverse events were observed in 93% of patients, with 11% experiencing grade 3 events and 1% experiencing a grade 4 event [49]. A dose reduction was necessary in 14% of patients, while 3% discontinued treatment due to adverse events [49]. Overall, treatment with Divarasib resulted in durable clinical responses across KRAS G12C-positive tumour with mostly low-grade adverse events.

D-1553, developed by InvestisBio, is an orally bioavailable KRAS G12C inhibitor which is currently under phase II study for NSCLC [50]. This compound has shown selective inhibition on KRAS G12C protein, which exerts potent anti-tumour effects on both *in vitro* and *in vivo* models without any effects on WT KRAS protein [50]. Mechanistically, D-1553 selectively inhibits ERK phosphorylation and downstream signalling pathways in NCI-H358 NSCLC cell line harbouring KRAS G12C [50]. In a phase I/II clinical trial for NSCLC and other solid tumours, recent findings indicate that D-1553 offered 40.5% ORR, 91.9% DCR and a median progression-free survival of 8.2 months in NSCLC patients carrying KRAS G12C mutations [51]. Treatment-related adverse events were reported by 94.9%, with 38.0% experiencing grade 3 or 4 events [51]. Altogether, these findings suggest that D-1553 might be a potentially effective and manageable treatment method for KRAS G12C-mutated NSCLC.

### Development of inhibitors for non-G12C KRAS mutants

In addition to KRAS G12C inhibitors, efforts have been made to develop inhibitors for KRAS G12D and G12V mutants. In 2017, Sakamoto *et al*. developed KRpep-2d, a cyclic 19-mer peptide selectively targeting both GDP-bound and GTP-bound KRAS G12D with high affinity [52]. It selectively binds to KRAS G12D and inhibits the exchange of GDP with GTP within this protein at an IC50 value of 1.6 nM [53]. KRpep-2d could selectively suppress cell growth in KRAS G12D—expressing cell lines [52]. However, a primary drawback of this peptide is its susceptibility to instability within the cellular reducing environment, leading to the cleavage of disulphide bonds [52]. To address this challenge, a derivative of KRpep-2d called KS-58 was developed to penetrate the target cell and inhibit interaction of mutant KRAS with its effector proteins [54]. Comparable anti-cancer effects of KS-58 have been observed in both subcutaneous and orthotopic PANC-1 mouse xenografts, indicating the therapeutic promise of this newly developed peptide for managing pancreatic cancer [54].

Another KRAS G12D inhibitors called MRTX1133 has been developed to target GTPase activity in KRAS G12D-driven lung cancer, pancreatic and colorectal adenocarcinoma models [55, 56]. This compound interacts with KRAS G12D with high affinity, inhibiting pERK, pS6 and cell viability in mutant cell lines ​​[55]. In xenograft models, MRTX1133 induces dose-dependent tumour regression and shows significant anti-tumour effects, including complete pERK inhibition in PDAC models [55]. In a phase I/II clinical trial for advanced solid tumours with KRAS G12D mutation, MRTX1133 is being evaluated for dose and regimen [30]. Additionally, Kemp *et al*. suggested that MRTX1133 may modulate the TME via alterations of immune cell landscape [56]. This dual mechanism of action could directly target tumour cells and reshape the TME, potentially enhancing responsiveness to immunotherapy [56].

Apart from the development of KRAS-mutant specific covalent inhibitor, efforts have been made to create non-covalent inhibitors aiming at sustained inhibition of mutant KRAS downstream signalling pathway. For instance, a recently developed monobody called 12VC1 exhibited up to 400 times higher specificity towards KRAS G12V and G12C mutations over WT KRAS [57]. This monobody could effectively block ERK phosphorylation, thus reducing cell proliferation rate in KRAS mutant cell lines such as H358, PATU8902, HPAF-II and A375 [57]. More importantly, 12VC1 did not shown any adverse effects on cell lines with WT KRAS expression [57] . For *In vivo* assessment, when used as a targeting ligand to create PROTAC-like degraders fused with E3 ubiquitin ligase subunit VHL, it led to a significant reduction in tumour size in mouse xenograft models [57] (Table 1).

**Table 1:** Clinical trials on inhibitors and siRNA for the treatment of KRAS-mutated cancers

Drug name	KRAS mutant target	Types of cancers	Clinical trial	Adverse outcomes	Ref
AMG510	G12C	NSCLC	NCT03600883 (I/II) NCT04303780 (III)	Diarrhoea, nausea, vomiting, fatigue, increased aminotransferase levels, hepatoxicity and cough	[39, 40, 116, 117]
MRTX849	G12C	CRC; NSCLC	NCT03785249 (I/II)	Diarrhoea, nausea, fatigue, vomiting, musculoskeletal pain, hepatotoxicity, renal impairment, dyspnea, edema, decreased appetite, cough, pneumonia, dizziness, constipation, abdominal pain and QTc interval prolongation	[41-44]
LY3537982	G12C	Advanced Solid Tumours	NCT04956640 (I)	Diarrhoea, constipation, fatigue, peripheral oedema, nausea, neutropenia	[48]
GDC-6036	G12C	Advanced and Metastatic Solid Tumours	NCT04449874 (I)	Rash, diarrhoea, nausea, vomiting, dry skin and paronychia	[49]
D-1553	G12C	Advanced and Metastatic Solid Tumours	NCT04585035 (I/II)	Diarrhoea, nausea, vomiting, rash, decreased appetite, liver function abnormalities and gastrointestinal events	[51]
MRTX1133	G12D	Advanced Solid Tumours	NCT05737706 (I/II)	-	[56]
12VC1	G12V	Pancreatic cancer	Pre-clinical	-	[57]
siG12D-LODER	G12D, G12C, G12V	Pancreatic cancer	NCT01676259 (II)	Diarrhoea and abdominal pain	[93]

## Lipid-based nanocarriers used in gene delivery

Despite the progress in KRAS G12C inhibitors, they fail to target other KRAS mutants that accounts for more frequent KRAS mutations in cancer patients. Additionally, it is important to note that the effectiveness of these inhibitors remains constrained due to the development of resistance mechanisms [2]. To overcome these challenges, gene therapy has emerged as a promising approach for targeting KRAS mutants by supressing [58]. For safe delivery of therapeutic gene agents, it is imperative to develop effective delivery systems capable of targeting specific cells, exhibiting low toxicity profiles and being scalable in manufacturing [59]. While viral vectors are commonly used for gene delivery, challenges such as immunogenic reactions and high manufacturing expenses impede their widespread application in clinical settings [60]. Among non-viral carriers, lipid-based nanoparticles (LNPs) have successfully demonstrated their capabilities as gene delivery systems. Their physicochemical properties, such as particle size, surface charge and lipid composition, can be systematically optimised to enhance stability, extend circulation time and improve the endosomal escape once upon cellular entry [61, 62]. Functionalising LNPs with tumour-specific ligands further enhances their selective uptake by cancer cells, minimising off-target effects and systemic toxicity [63]. Recent advances in formulation techniques, including the incorporation of stabilising agents and the use of ionizable lipids, have significantly improved the structural integrity, shelf-life and scalability of these nanocarriers, paving the way for clinical translation [64]. Therefore, in this section, we will particularly discuss the LNPs including liposomes, solid lipid nanoparticles (SLNs) and lipid-polymer hybrid nanoparticles (LPNs), as potential vehicles for gene therapy in cancer treatment.

### Liposome

**Fig. 3:**
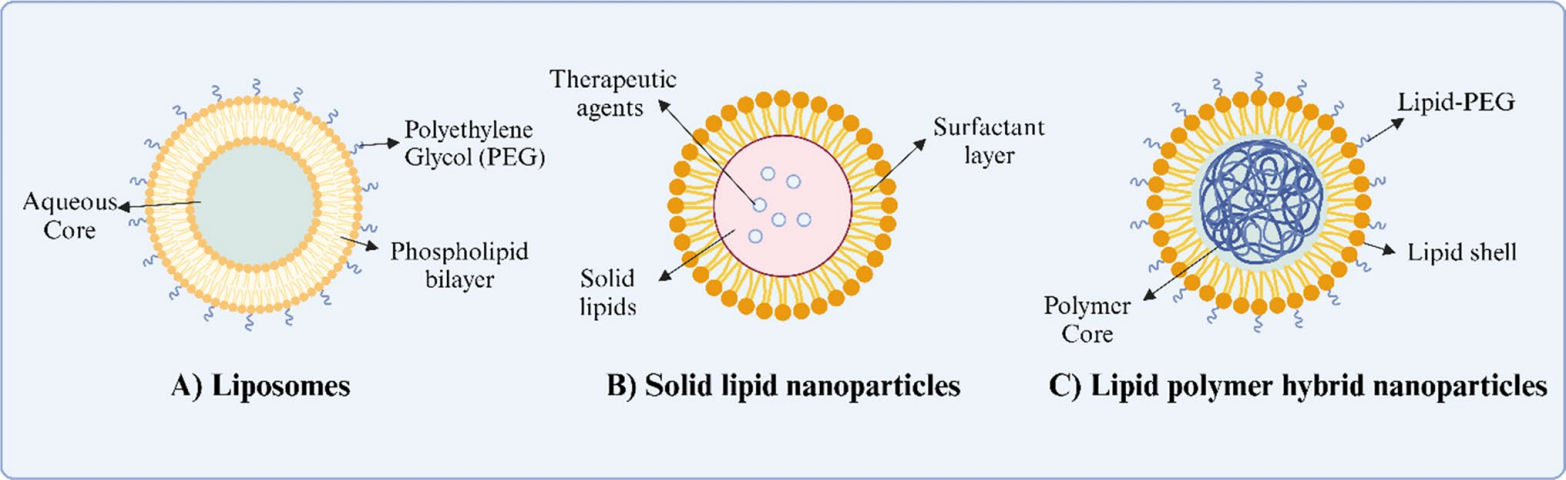
Different types of lipid-based nanoparticles. **A** Liposomes are spherical vesicles characterized by one or multiple phospholipid bilayers organised concentrically around aqueous cores. **B** SLNs are defined by a solid lipid core surrounded by a layer of surfactants within a water-based dispersion. **C** LPNs integrate both lipid- and polymer-based elements, featuring a polymer core surrounded by a lipid/lipid-PEG layer

Liposomes are nanosized lipid carriers formed by self-assembling lipids, typically comprising one or multiple phospholipid bilayers arranged concentrically around a discrete aqueous core [65]. This structure allows liposomes to transport hydrophilic molecules within their aqueous core and hydrophobic molecules in the lipid bilayer (Fig. 3A) [66]. Liposomes have been extensively utilised for gene delivery due to their unique advantages, such as high biocompatibility, ability to carry large drug payload, controlled release and large-scale production feasibility [67]. They can be easily modified to enhance the therapeutic efficacy and reduce the immunogenicity effect, such as refining liposomes with ionisable cationic lipids to facilitate membrane fusion, employing targeted liposomes with ligands attached to their surface, or applying biocompatible polymer coating like PEG to evade the immune system [68, 69].

One research team has recently developed a liposome-based platform for the delivery of CRISPR/Cas9 system targeting HPV16 E6/E7 gene in cervical cancer [70]. By using gRNA/Cas9 system, *in vitro* findings revealed a reduction of over 90% in E6 and E7 gene expression and an approximate 80% enhancement of HMGB1 and ATP levels indicating the antitumour immune activation. Further *in vivo* results demonstrated that this HPV-gRNA/Cas9-liposome induced apoptosis in tumour cell, inhibited tumour growth, activated antitumour immune responses and reversed the immunosuppressive tumour microenvironment in the mouse model bearing cervical cancer. Additionally, combining HPV-gRNA/Cas9-liposome treatment with anti-PD-1 therapy exhibited the enhanced antitumour efficacy, with an approximately two-fold increase in the HMGB1 and ATP level compared to either treatment alone. These findings indicated the effectiveness of the liposomes as gene delivery systems, highlighting their potential in gene therapy applications, especially when utilised in combination with other therapy modalities.

### Solid lipid nanoparticles

SLNs are characterized by a solid lipid core enveloped by a layer of surfactants within an aqueous dispersion, displaying dimensions ranging approximately from 50 to 1000 nm (Fig. 3B) [71, 72]. In the SLNs, the active substance can be incorporated into the rigid core of lipid matrix [73]. Similar to liposomes, SLNs offer several advantages, such as the ability to protect active ingredients from degradation and the potential to regulate the release profile of these ingredients [74]. Moreover, incorporating cationic lipids or attaching functional molecules like antibodies or ligands to SLN surface will enhance their cellular uptake activity and specific targeting capability [73]. For example, one study reported the use of cationic SLNs (cSLNs) employing didodecyldimethylammonium (DDAB) cationic lipid to deliver siRNA targeting KDM4A and EphA2 genes, which are both commonly overexpressed in cancer cells [75]. This nanocarrier exhibited approximately 30% enhanced cellular uptake activity in prostate cancer cells (DU145 and PC-3) compared to commercial transfection reagents (Dharmafect 2). Additionally, this study showed that co-administration of cSLNs/siRNA with JIB-04, a histone lysine demethylase inhibitor, effectively suppressed migratory activity and reduced colony intensity by over 80% in PC-3 cells. This combined treatment achieved a roughly 35% reduction in KDM4A mRNA expression and a 50% reduction in EphA2 mRNA expression compared to the administration of cSLNs/siRNA alone. However, SLNs still face the major limitations such as low loading efficiency, short shelf life and poor long-term drug retention [76–78]. These challenges may vary depending on factors such as the formulation, the cargo being loaded and storage conditions. Therefore, further optimisation of these factors is essential to ensure the capability and effectiveness of SLNs.

### Lipid-polymer hybrid nanoparticles

LPNs comprise a polymer core surrounded by an inner lipid layer and an outer lipid-PEG layer (Fig. 3C) [79, 80]. The biodegradable core often contains polymers like polylactic-co-glycolic acid (PLGA), which can enhance encapsulation rate of therapeutic agents [81]. Meanwhile, their inner lipid layer decreases the diffusion of encapsulated content and slows down the polymer degradation rate, facilitating sustained release of the content [80]. The outer lipid-PEG layer protects LPNs, preventing the immune system and prolonging *in vivo* circulation time [80]. These unique compositions make LPNs an ideal delivery platform for gene therapy. A recent study developed a LPN “particle-in-particle” system to deliver plasmid DNA and mRNA [82]. The optimised formulation exhibited enhanced transfection efficacy compared with Pfizer/BioNTech COVID-19 vaccine formulation, achieving a *in vitro* transfection efficiency of approximately 95% for the GFP gene at 96 h post-transfection. Moreover, this system effectively triggered spike-specific antibodies and Th1-biased T-cell immune response in a BALB/c mouse model. Despite their promising efficacy, challenges associated with LPNs still persist, including toxicity of polymer components and inconsistencies in size and shape [72]. Therefore, ongoing research efforts are focused on overcoming these obstacles to enhance the utility of LPNs across various gene therapy applications.

## Application of gene therapy targeting mutant KRAS in cancers

As aforementioned, most KRAS inhibitor drugs target KRAS G12C, the predominant KRAS-mutant type in NSCLS [83]. However, they do not effectively target other KRAS mutants, such as KRAS G12D, KRAS G12S and KRAS G13, which are more common in other types of cancer [84]. This section investigates the potential of employing gene silencing/editing strategies, such as small interfering RNAs (siRNAs), microRNAs (miRNAs) and CRISPR-Cas9, to specifically target other KRAS mutants. It focuses on utilising polymers, LNP and viral vectors as delivery vehicles for gene therapy in this context.

### siRNA-based approach

siRNAs are small, double-stranded RNA molecules of 21 to 23 base pairs, enabling precise gene silencing through RNA interference by binding to one mRNA of targeted gene [85]. This binding blocks the translation process of its encoded protein, thereby inhibiting aberrant cell signalling pathway activated by gene mutation and inducing apoptosis [86]. Effective siRNA-based therapy need to meet several criteria. Firstly, the siRNA sequence requires optimization to ensure specificity for binding to the RNA-induced silencing complex and the target mRNA, thus minimising off-target effects [87]. Secondly, for therapeutic efficacy, siRNAs must successfully reach the target cells or tissues [88]. In addition, it is crucial for siRNA agents to be stable, preventing degradation by RNases present either in serum or within the endocytic compartments of cells [89]. To address these challenges, two different strategies have been investigated by either chemical modification of the siRNA molecule itself or incorporating it into a delivery vehicle [90, 91]. These approaches provide protection from degradation, minimise off-target effects and facilitate targeted delivery of siRNA to specific cells or tissues.

Several studies have investigated the utility of siRNA to target KRAS mutants in various cancer models (Table 2). For example, one study developed nanovesicles consisting of cyclic RGD peptide-modified polymersomes loaded with siRNA (cRGD-BCP-siKRAS) to target KRAS G12D mutation [63]. These nanoparticles achieved a remarkable 90% gene knockdown efficacy at a siRNA dose of 3 mg/kg in a mouse model harbouring the KRAS G12D mutation. This led to significant inhibition in tumour growth, with 40% of mice achieving complete regression. These findings indicate that cRGD-BCP-siKRAS could hold promise as a treatment for KRAS G12D-mutated pancreatic cancer. Perepelyuk *et al*. reported the *in vivo* therapeutic efficacy of siRNA targeting KRAS G12S mutation in non-small-cell lung cancer (NSCLC) [92]. In this study, authors developed hybrid nanoparticles consisting of a block copolymer and human immunoglobulin G incorporating siRNA. These siRNA-loaded nanoparticles effectively inhibited A549 cancer cell proliferation. Furthermore, *in vivo* findings demonstrated their excellent antitumour effect in a metastatic murine model. A significant decrease of 60% in KRAS G12S expression was observed in a mouse model, resulting in regression of tumour burden while causing minimal toxicity to healthy tissues. These findings suggest the potential of siRNA therapy as a precise and potent treatment for NSCLC harbouring the KRAS G12S mutation [92].

**Table 2:** Preclinical studies on gene therapy targeting mutant KRAS genes

Therapeutic gene	Delivery systems	KRAS mutant target	*In vitro* results	*In vivo* results	Ref
siRNA	cRGD modified bioresponsive chimeric polymersomes (cRGD-BCP)	G12D	siKRAS delivered by cRGD-BCP knowdown KRAS G12D by 90% in PANC-1 pancreatic cancer cells	Effective tumour targeting, tumour growth inhibition and prolonged survival in PANC-1 tumour-bearing mice. 40% of the mice achieved complete regression and increase in median survival time	[63]
siRNA	siG12D-LODER (miniature biodegradable polymeric matrix)	G12D	Effective KRAS G12D silencing and inhibited cell proliferation in PDAC cells	No tumour progression, with the majority (10/12) demonstrating stable disease, median overall survival was reported as 15.12 months, and the 18-month survival rate was 38.5% with a single dosage of siG12D-LODER™ without repeat dosing	[62, 93]
siRNA	tLyp-1 conjugated LNP	pan-KRAS	Significant reduction in KRAS expression CFPAC-1 pancreatic cancer cells	tLyp-1 tagged LNPs had an enhanced accumulation in the tumour compared to non-targeted LNPs. 50% reduction in tumour growth was observed for treatment using tLyp-1 LNP combined with gemcitabine in athymic CD1 nude mice with subcutaneously injected CFPAC-1 human pancreatic cancer cells	[61]
miRNA	Neutral lipid emulsion (NLE)	G12D	Reduction of over 95% in luminescence, indicating effective silencing of the luciferase gene in H460-luc non-small cell lung cancer cells	Treatment resulted in 60% reduction of tumour growth and metastasis in autochthonous KRAS G12D transgenic mouse model of lung cancer	[118]
miRNA	Lipofectamine 3000	G12S	Significantly reduced the expression of KRAS G12S gene in the A549 NSCLC cells. The specificity of amiR-KS3 was evident that it did not affect expression of wild-type KRAS in other cell lines such as H1299 and H292	Reduced tumour volume after intratumoural injection of amiR-KS3; Reduced necrotic areas in xenograft mouse model	[106]
CRISPR Cas9	Viral vectors (adenoviral and lentiviral)	G12S	Inhibit the proliferation of A549 cells (lung adenocarcinoma cells with KRAS G12S mutant allele), with 77% editing efficiency for the SpCas9-sgG12S system. No significant effect was observed in H2228 cells (lung adenocarcinoma cell line without G12S mutation), indicating the specificity of the systems for the mutant allele	Local adenoviral injections resulted in significant tumour regression *in vivo*. Specifically, there was a 46% reduction in tumour volume with the SpCas9 system and a 15.6% reduction with the dCas9-KRAB system in A549-bearing mice. These effects were not observed in H2228-engrafted mice, demonstrating the specificity of the treatment for the KRAS G12S mutation	[112]

In addition to advancements in preclinical investigations, clinical trials were initiated to explore siRNA-based therapy targeting the KRAS mutant (G12D) [62]. For instance, siG12D-LODER, a co-polymer composed of poly lactic-co-glycolic acid (PLGA) encapsulating siRNA targeting KRAS G12D, was investigated in pancreatic ductal adenocarcinoma (PDAC) patients [62]. In a phase 1/2a study involving patients with non-operable locally advanced pancreatic cancer (LAPC), single administration of three increased doses of siG12D-LODER alongside standard of care chemotherapy (Gemicitabine or Oxaliplatin + Irinotecan + Fluorouracil or Gemicitabine + Erlotinib + Oxaliplatin) was given to patients [62]. Promising outcomes were observed in this trial, with the majority of patients achieving stable disease (with grade 1 or 2 adverse events), no tumour progression and a median overall survival of 15.12 months. Additionally, the 18-month survival rate of 38.5% was achieved among these patients with no dose limiting toxicity events [62]. An ongoing phase 2 trial evaluates the efficacy of siG12D-LODER at a dosage of 2.8 mg over 12 weeks in patients with LAPC, in conjunction with chemotherapy drugs such as Gemcitabine + nab-Paclitaxel or Folfirinox or modified Folfirinox, aiming to determine the response rate of siG12D-LODER in these patients [93]. These findings suggest that siG12D-LODER could serve as a promising adjunctive therapy for the treatment of pancreatic cancer.

**Fig. 4:**
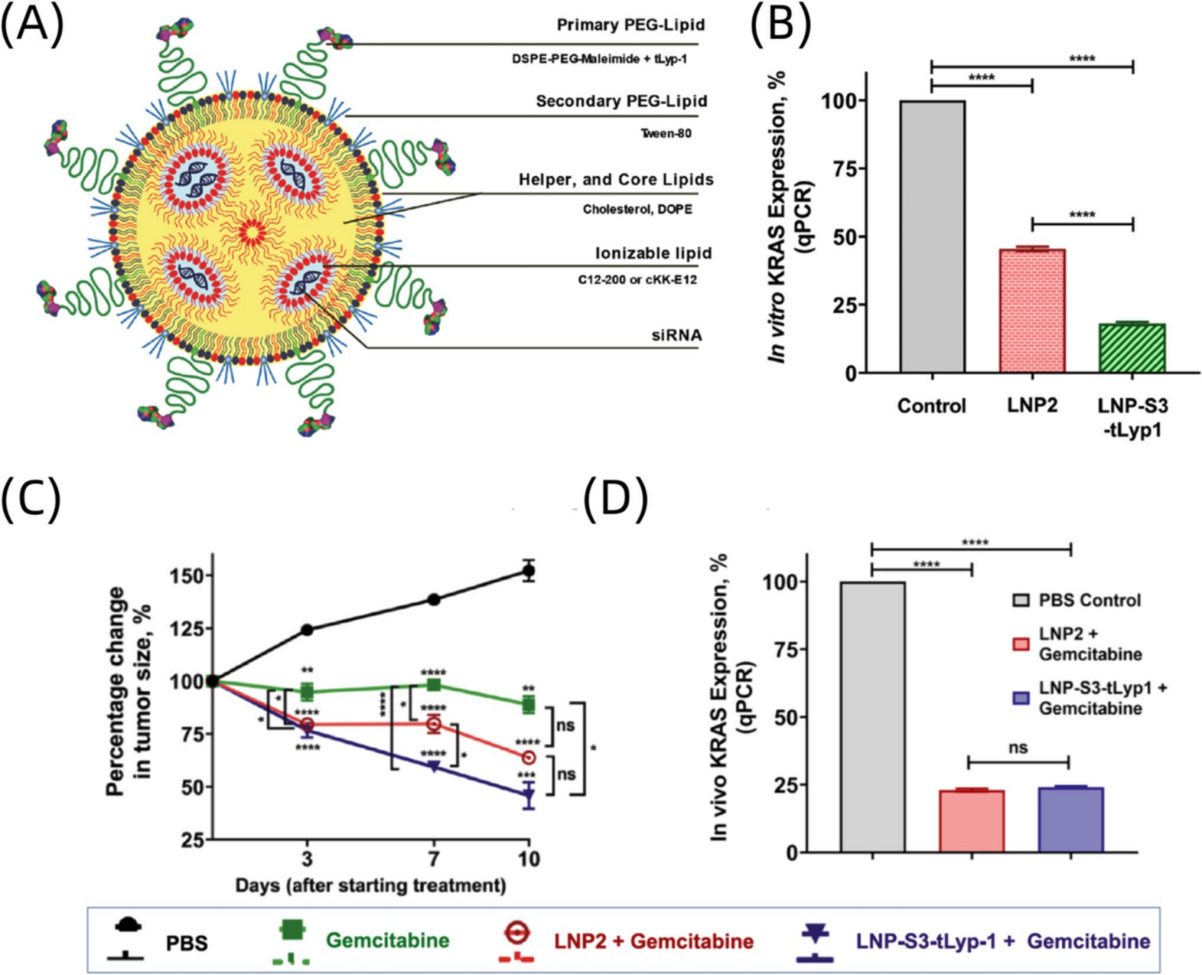
*In vivo* therapeutic effect of LNP-mediated siRNA on pancreatic cancer with pan-KRAS. **A** Schematic illustration of targeted LNP encapsulating siRNA. **B** *In vitro* KRAS expression measured by qPCR. **C** Percentage change in tumour size. **D** *In vivo* KRAS expression measured by qPCR. Figure adapted from Anthiya *et al*. (2023) [61]

In addition to targeting specific subtypes of KRAS mutations, new research directions have shifted towards developing siRNA therapies capable of simultaneously inhibiting multiple types of KRAS mutations [61]. For example, a custom siRNA, EFTX-D1, has shown specificity in suppressing the expression of KRAS mutations in codon 12 and codon 13 without affecting WT KRAS in various lung cancer cell lines [94]. In another study, Anthiya *et al*. employed a LNP delivery system loaded with siRNA targeting pan-KRAS [61]. The surface of this nanoparticle was conjugated with the tLyp-1 peptide (Fig. 4A), enhancing its targeting capability to pancreatic cancer cells. After the treatment with siRNA-LNPs, significant reduction in KRAS mRNA expression was observed in CFPAC-1 pancreatic cancer cells (Fig. 4B). Additionally, *in vivo* results showed a notable 50% reduction in tumour size compared to other groups (Fig. 4C). Tumours collected after treatment exhibited a significant reduction in KRAS expression (Fig. 4D). Taken together, these findings suggested that targeted siRNA-LNPs hold promise for enhancing tumour targeting and effectiveness of siRNA therapy at both *in vitro* and *In vivo* setting.

### miRNA-based approach

miRNAs are single-stranded, non-coding RNAs around 20 to 24 nucleotides long, which negatively regulate the expression of multiple mRNA targets to induce translational repression [95, 96]. Due to their ability to regulate the expression of multiple genes, each miRNA can efficiently coordinate various cellular pathways and processes related to cancer cellular growth and proliferation [97, 98]. Similar to siRNA, miRNA encounters the challenges such as off-target effects and instability [97]. Mitigating strategies including chemical modification of miRNA structure and incorporating it into a delivery system can be used to enhance the specificity and efficacy.

The applications of miRNAs, has shown promising result in targeting mutant KRAS inhibition in several types of cancers (Table 2) [99, 100]. So far, miRNAs such as miRNA *let-7*, miR-18a-3p, miR-29b-3p, miR-30b, miR-126-5p, miR-143-3p,miR-143-3p and miR-155-5p have been identified as KRAS regulators in different types of cancers [101]. The study reported by Stahlhut *et al*. has revealed that the aforementioned miRNAs are often downregulated or lost in cancer and restoration of these miRNAs can supress tumour growth by inhibiting these oncogenic pathways [102, 103]. Based on this observation, the combination of miRNA *let-7* and miR-34 has been used to sensitise erlotinib chemotherapy in NSCLC cell line carrying KRAS G12D mutation [104]. This is exemplified in the work undertaken by Trang *et al*. where they found that the systemic delivery of miRNA *let-7* or miR-34 using neutral LNPs led to a significant reduction in tumour size in a mouse model of NSCLC with KRAS G12D mutation [105]. This phenotype was accompanied by decreased cell proliferation and increased tumour apoptosis in mRNA-treated mice. In short, this study highlights the safety profile of utilising neutral LNP systems, as evidenced by the absence of preferential miRNA accumulation in the liver and the lack of induction of non-specific immune responses. Additionally, Acunzo *et al*. used Lipofectamine 3000 as a transfection reagent to deliver artificial miRNA called amiR-KS3 targeting KRAS G12S in NSCLC cell lines. This delivery method has effectively inhibited KRAS mutation without affecting the wild-type KRAS and reduced tumour cell growth and migration in both *in vitro* and *In vivo* models of NSCLC [106].

### CRISPR-based approach

The CRISPR/Cas9 system consists of two critical components, the Cas9 enzyme and the guide RNA (gRNA). The editing mechanism involves the integration of a DNA fragment from an invading pathogen into the CRISPR locus. Upon subsequent infection, this integrated DNA is transcribed and processed into mature guide RNAs (gRNAs). These gRNAs direct the Cas9 protein to distinct genomic locations to create double-strand breaks (DSBs), with the requirement that the 3′ end of the target sequence must have an NGG protospacer adjacent motif (PAM) [107]. The resultant DSBs then trigger DNA repair by non-homologous end joining (NHEJ) or homology-directed repair (HDR), facilitating precise genome editing [108]. Genome editing via the CRISPR/Cas9 method allows for precise alterations of genetic sequences, which is crucial for uncovering genes implicated in cancer development and correcting mutations that cause cancer [109].

This gene editing tool has also been utilised in targeting mutant KRAS-driven cancers [110, 111]. For instance, recent studies reported the therapeutic efficacy of two CRISPR systems delivered by viral vectors, SpCas9 and dCas9-KRAB, in mouse models of NSCLC with KRAS G12S mutation [112, 113]. Particularly the SpCas9 system exhibited significant efficacy, resulting in a 46% reduction in tumour volume and a 30% decrease in tumour weight [112]. Conversely, the dCas9-KRAB system, operating by regulating gene transcription, exhibited a modest yet noteworthy decrease with a 15.6% reduction in tumour volume [112]. It is important to note that the CRISPR technique faces several challenges, including the potential off-target effects, immunogenicity risks and ethical considerations [114]. Additionally, the considerable molecular weight of Cas9 protein (160 kDa, 4300 bases) poses limitations on its delivery of both viral and non-viral vectors [114].

Beyond the standalone application of gene therapy described earlier, combining siRNA and CRISPR-based gene therapy approaches with immunotherapies or chemotherapies holds significant promise to enhance anti-tumour responses and overcome drug resistance. For instance, silencing KRAS mutations using siRNA has been shown to enhance tumour cell sensitivity to chemotherapeutic agents such as gemcitabine, leading to reduced tumour progression in pancreatic cancer models [61]. Furthermore, KRAS mutations have been linked to the upregulation of the immune checkpoint molecule PD-L1, suggesting that CRISPR-mediated knockout of PD-1 or PD-L1 could improve the efficacy of immunotherapies in KRAS-mutant lung cancers [115]. Taken together, these findings highlight the promise of combining gene-silencing or gene-editing strategies with established immunotherapeutic or chemotherapeutic regimens to achieve more durable and effective cancer control.

In summary, siRNA, miRNA and CRISPR-based therapies each exhibit distinct advantages and limitations (Table 3). siRNA offers relatively high specificity but faces challenges with delivery and transient effects. miRNA’s ability to target broader signalling pathways can be advantageous but raises concerns about unintended impacts on non-target genes. CRISPR provides permanent genomic edits but involves significant ethical and safety considerations. Understanding these differences is essential for selecting the most suitable therapeutic strategy in the context of KRAS-driven cancers.

**Table 3:** Comparison of mechanisms, advantages and limitations of siRNA, miRNA and CRISPR-based therapies

	Mechanism	Advantage	Challenge
siRNA	Short double-stranded RNA binds to target mRNA, leading to degradation and preventing translation into protein	High specificity for single gene silencing; relatively simple design; potential for transient effects, making it safer for temporary therapies	Limited stability and delivery efficiency; potential for off-target effects; transient silencing may require repeated treatments
miRNA	Endogenous single-stranded RNA regulates gene expression by binding to multiple message RNAs, influencing signalling networks	Ability to regulate entire signalling networks; natural integration in cellular pathways; broad therapeutic potential	Challenges in achieving precise modulation; off-target effects in non-cancer related pathways; broad action may cause unintended impacts
CRISPR	The RNA-guided Cas9 endonuclease induces double-strand breaks in DNA, subsequently activating DNA repair pathways such as non-homologous end joining or homology-directed repair, resulting in permanent gene editing	Permanent genetic modifications; high precision; potential to correct genetic mutations directly	Off-target edits; potential immunogenicity; ethical concerns over permanent genome alterations; delivery remains a challenge

## Conclusions

Gene therapy targeting KRAS mutations shows promising data in both preclinical and clinical stage, but overcoming key challenges is crucial for successful translation into clinical practice. While miRNAs have been explored for their potential in targeting KRAS gene mutations, they may not serve as optimal direct therapeutics for treating KRAS-mutant cancers. This was because they may affect multiple cellular pathways by targeting numerous mRNA targets [119]. Moreover, individual miRNAs may exert opposing effects in different tissues, potentially leading to systemic off-target effects in clinical trials [120]. siRNA has emerged as a promising candidate with clinical trials demonstrating positive outcomes. However, the applications of siRNA therapeutics remains challenging. For instance, the short lifespan of siRNA molecules can be addressed by modifying their structure [88]. Another major challenge lies in efficiently delivering siRNA agents to targeted cancer cells within the body, as off-target effects inherent to siRNA can compromise both therapeutic efficacy and safety [121]. To address this issue, researchers have focused on the engineering of delivery systems specifically for targeted delivery to cancer cells. By modifying the surface of LNPs with targeting ligands, such as antibodies and peptides, they can selectively bind to receptors overexpressed on cancer cell surface, enhancing specificity and reducing off-target effects [122]. Additionally nanocarriers can be designed to respond to the acidic tumour microenvironment, enabling siRNA release at the tumour site [123].

The newly-emerged CRISPR prime editing technology holds tremendous promise as a highly precise editing tool for altering KRAS mutations while preserving WT KRAS functions within the targeted cancer cells without the need for double-strand breaks like the conventional CRISPR-Cas9 system [124–126]. Significantly, this editing tool delivered by LNPs has demonstrated minimal off-target effects in organoids and mouse models, thus holding significant therapeutic potentials for KRAS-mutant cancer treatment [127, 128].

In conclusion, siRNA and CRISPR appear to be the most suitable approaches for editing mutant KRAS. Further research is necessary to enhance the targeting specificity of these tools and optimise the nanocarriers like LNPs as safe and efficient gene delivery vehicles. This will enable the development of more effective and precise treatments for cancers associated with KRAS mutations.

## Data Availability

No datasets were generated or analysed during the current study.
